# Hitting the target: fragment screening with acoustic *in situ* co-crystallization of proteins plus fragment libraries on pin-mounted data-collection micromeshes

**DOI:** 10.1107/S1399004713034603

**Published:** 2014-04-30

**Authors:** Xingyu Yin, Alexander Scalia, Ludmila Leroy, Christina M. Cuttitta, Gina M. Polizzo, Daniel L. Ericson, Christian G. Roessler, Olven Campos, Millie Y. Ma, Rakhi Agarwal, Rick Jackimowicz, Marc Allaire, Allen M. Orville, Robert M. Sweet, Alexei S. Soares

**Affiliations:** a Office of Educational Programs, Brookhaven National Laboratory, Upton, NY 11973-5000, USA; b Department of Biochemistry and Cell Biology, Stony Brook University, NY 11794-5215, USA; c Nanjing University, Nanjing, Jiangsu, People’s Republic of China; d Department of Biological Sciences, Binghamton University, 4400 Vestal Parkway East, NY 13902, USA; e CAPES Foundation, Ministry of Education of Brazil, 70040-020 Brasilia-DF, Brazil; f Universidade Federal de Minas Gerais, 6627 Av. Antonio Carlos, 31270-901 Belo Horizonte-MG, Brazil; g Center for Developmental Neuroscience and Department of Biology, College of Staten Island, The City University of New York, 2800 Victory Boulevard, Staten Island, NY 10314, USA; h St Joseph’s College, 155 West Roe Boulevard, East Patchogue, NY 11772, USA; i Department of Biomedical Engineering, University at Buffalo, SUNY, 12 Capen Hall, Buffalo, NY 14260, USA; j Photon Sciences Directorate, Brookhaven National Laboratory, Upton, NY 11973-5000, USA; k Department of Biological Science, Florida Atlantic University, 777 Glades Road, Boca Raton, FL 33414, USA; l Comsewogue High School, 565 Bicycle Path, Port Jefferson Station, NY 11776, USA; m Biosciences Department, Brookhaven National Laboratory, Upton, NY 11973-5000, USA

**Keywords:** *in situ* X-ray data collection, acoustic droplet ejection, fragment screening, drug discovery, chemical biology, protein crystallization, synchrotron radiation

## Abstract

Acoustic droplet ejection (ADE) is a powerful technology that supports crystallographic applications such as growing, improving and manipulating protein crystals. A fragment-screening strategy is described that uses ADE to co-crystallize proteins with fragment libraries directly on MiTeGen MicroMeshes. Co-crystallization trials can be prepared rapidly and economically. The high speed of specimen preparation and the low consumption of fragment and protein allow the use of individual rather than pooled fragments. The Echo 550 liquid-handling instrument (Labcyte Inc., Sunnyvale, California, USA) generates droplets with accurate trajectories, which allows multiple co-crystallization experiments to be discretely positioned on a single data-collection micromesh. This accuracy also allows all components to be transferred through small apertures. Consequently, the crystallization tray is in equilibrium with the reservoir before, during and after the transfer of protein, precipitant and fragment to the micromesh on which crystallization will occur. This strict control of the specimen environment means that the crystallography experiments remain identical as the working volumes are decreased from the few microlitres level to the few nanolitres level. Using this system, lysozyme, thermolysin, trypsin and stachydrine demethylase crystals were co-crystallized with a small 33-compound mini-library to search for fragment hits. This technology pushes towards a much faster, more automated and more flexible strategy for structure-based drug discovery using as little as 2.5 nl of each major component.

## Abstract 2

A method is presented for screening fragment libraries using acoustic droplet ejection to co-crystallize proteins and chemicals directly on micromeshes with as little as 2.5 nl of each component. This method was used to identify previously unreported fragments that bind to lysozyme, thermolysin, and trypsin.

## 1. Introduction   

Structure-based drug discovery using X-ray crystallography as a primary fragment-screening tool (Chilingaryan *et al.*, 2012 ▶) allows simultaneous structural characterization of each binding site, including allosteric sites (Bauman *et al.*, 2013 ▶), and the immediate capacity to improve the potency or pharmaceutical characteristics of the fragment hit (Edwards *et al.*, 2007 ▶). Fragment strategies attempt to screen a low-molecular-weight library and subsequently improve the initial hit to achieve a tight-binding lead compound (Erlanson *et al.*, 2004 ▶). In co-crystallization searches, the consumption of 1–10 µg protein and ∼100 nl of chemical per screened condition has been reported (Klages *et al.*, 2007 ▶; Rich & Myszka, 2004 ▶; Erlanson *et al.*, 2004 ▶). The optimum throughput for the acquisition of X-­ray data is ∼1 min per screened fragment (limited by the maximum speed of robotic automounters; Cork *et al.*, 2006 ▶), while a realistic throughput is a slower ∼4 min per screened condition (Wasserman *et al.*, 2011 ▶). Crystallization is often automated, but crystal harvesting is frequently manual and laborious, although it can also be automated (Cipriani *et al.*, 2012 ▶; Soares *et al.*, 2011 ▶; Viola *et al.*, 2007 ▶). The throughput rate can be increased by grouping fragments into cocktails of chemically compatible compounds, which are often structurally diverse to facilitate identification in the electron density (non-diverse compounds may be used in some cases; Nicholls *et al.*, 2010 ▶). When a binding event is observed, the cocktail is deconvoluted either using the electron density or by individually screening each cocktail member during a second-pass experiment (Spurlino, 2011 ▶). Existing fragment-screening strategies work well in commercial applications, where the cost of fragment libraries and the availability of purified protein are not limiting factors. However, the resource boundaries that constrain most academic efforts will not stretch enough to screen a typical 2000-fragment library using 1 µl protein and 4 min of synchrotron beam time per specimen. A faster and more efficient strategy is needed.

Here, we report a fully automated system for using acoustic droplet ejection to co-crystallize a protein of interest with a fragment library using 2.5–40 nl purified protein (0.025–0.400 µg at 10 mg ml^−1^) and 2.5–10 nl fragment compound per screened condition. Crystals are grown directly on data-collection media, such as MiTeGen MicroMeshes, and consequently no looping or mounting is needed. The specimen preparation rate is ∼60 per minute (including protein, precipitant and fragment). Using a conventional cryogenic automounter system, a shutter-less data-acquisition rate of ten screened conditions per minute is achieved by positioning multiple crystal and fragment pairs on each data-collection micromesh (so that each robotic automounter cycle inserts ten discrete experimental conditions for testing). This sustained rate of ten screened conditions per minute will approach the maximum data-acquisition speed that can be supported by the available X-ray intensity at third-generation synchrotrons. For example, if the full NSLS II X-ray fan is focused into a 20 µm square beam at the AMX beamline (currently under construction), protein crystals will be fully exposed to their radiation-dose limit (2 × 10^7^ Gy) in 2 s (Hodgson *et al.*, 2009 ▶). This high rate of sustained data acquisition will allow structure-based fragment screening without having to group chemicals into cocktails, thus mitigating the effects of a high aggregate fragment concentration on protein stability and crystallization (Boyd & Kloe, 2010 ▶; Baurin *et al.*, 2004 ▶), avoiding the possibility of inter-fragment interactions (Drinkwater *et al.*, 2010 ▶; Nair *et al.*, 2012 ▶) and avoiding the need for a deconvolution strategy to differentiate between the fragments in each cocktail (Nicholls *et al.*, 2010 ▶).

**Figure 1:**
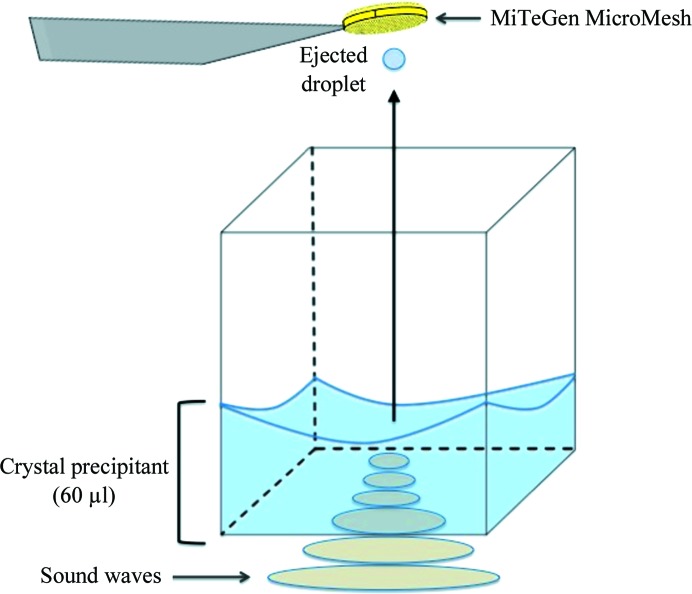
Acoustic droplet ejection. Acoustic droplet ejection (ADE) uses sound energy to transfer variable micro-droplets (*e.g.* nanolitres or picolitres) of solution (protein, precipitant, fragments *etc.*) from a crystallization well, through a short air column (∼1 cm) to data-collection media. Sound-wave energy from the transducer is channeled to the focal point (*i.e.* ejection zone), displacing the surface where a controlled ejection occurs. Droplet size is governed by the wavelength of the sound emitted and this proportionality yields accurate ejected volumes. In this work, an Echo 550 liquid handler was used to co-crystallize proteins, precipitants and fragments *in situ* directly on MiTeGen MicroMeshes by vapour diffusion. The Echo 550 does not use frequency changes to transfer different volumes. Instead, it uses a fixed-frequency sound pulse to transfer each component in 2.5 nl increments.

Acoustic droplet ejection (ADE) has a demonstrated utility for growing protein crystals (Villaseñor *et al.*, 2012 ▶), improving the quality of protein crystals (Villaseñor *et al.*, 2010 ▶) and manipulating protein crystals (Soares *et al.*, 2011 ▶). This method uses a sound pulse to transfer momentum to a liquid (or suspended solid). The liquid is then propelled out of the source location, through a short air column and onto an arbitrary destination (Ellson *et al.*, 2003 ▶; Fig. 1 ▶) with a published volumetric accuracy of 5% and a measured trajectory precision of 1.3° (data not shown; larger for some fluids). The high trajectory accuracy enables a ‘drop-on-drop’ capability which supports the combination of distinct components from different source wells onto the same destination location. Our group has demonstrated that a high ‘drop-on-drop’ accuracy is sustained across a wide variety of commercially available crystallization conditions and cryoprotectants (Cuttitta *et al.*, 2014 ▶). The transferred volume of the liquid is governed by the frequency of the sound (a typical working volume is 2.5 nl), and the velocity is determined by the amplitude of the sound (a typical ejection velocity is 1 m s^−1^). To eject larger volumes, the Echo 550 liquid-handling instrument does not modulate the frequency of the sound, but rather emits multiple sound pulses to build up the desired volume in 2.5 nl increments.

Villasenor and coworkers have suggested that acoustic methods might be used for structure-based drug discovery by co-crystallizing proteins and fragments using a shared reservoir on a conventional crystallization plate (Villaseñor *et al.*, 2012 ▶). Acoustic methods are an attractive choice for micro-crystallization for several reasons. ADE is an automated technique that is independent of operator skill. It is physically gentle, with no tips or tubes that may leach chemicals, cause cross-contamination between specimens (McDonald *et al.*, 2008 ▶) or damage crystals. Transfers have high accuracy even at very low volume (2.5 nl), with zero loss of specimen since there are no tips or tubes that liquids can adhere to (zero lost volume per transfer). The inaccessible volume at the bottom of each well is very small (4 µl dead volume; Harris *et al.*, 2008 ▶) and can readily be reduced even further (Cuttitta *et al.*, 2014 ▶). Specimen transfer is fast (2.33 ± 0.04 mounts per second to multiple destinations, 500 mounts per second between fixed locations; data not shown), which reduces specimen preparation time, and consequently also reduces the time during which specimens are exposed to atmospheric dehydration. Dehydration effects can be virtually eliminated by transferring all proteins, precipitants and fragment components through small apertures in the destination plate (called the pin platform box; see §2.1). Apertures can also be used in the source plate. Fragments solvated in DMSO are hygroscopic and will rapidly swell with incorporated water when exposed to atmospheric humidity. This problem is prevented by keeping the source plate covered with a plastic seal and by transferring DMSO-solvated fragments through apertures in the plastic seal.

When crystals are grown directly on data-collection media and robotically cryocooled, all steps in the fragment-screening process become fully automated. One consequence of full automation is that all metadata are machine-generated and can potentially be automatically deposited into a common database. Robust integration of automated specimen preparation with the X-ray data-acquisition database will facilitate implementation of a workflow-management program to simplify complex drug-discovery projects (Tsai *et al.*, 2013 ▶). Conveniently, no time is wasted on laboriously fishing protein crystals out of hanging or sitting drops. The equipment is operated through an intuitive GUI with minimal training. The entire fragment-library screening process is keyboard-driven and compatible with remote operation. As an illustration of the robust simplicity of this system, the experimental work for this project was largely performed by a team of diligent undergraduate students during a ten-week summer internship program.

## 2. Methods   

**Figure 2:**
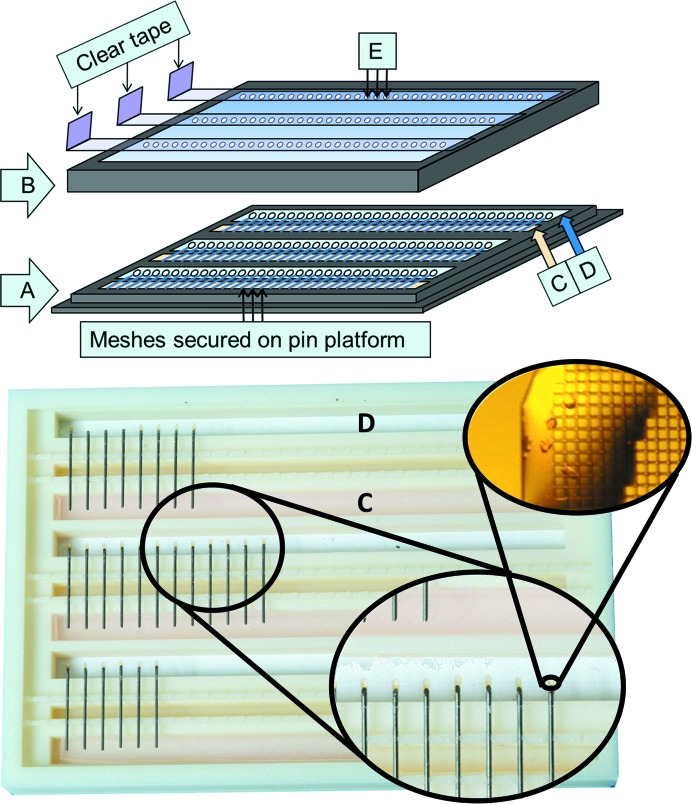
Pin platform box. *In situ* vapor-diffusion co-crystallization is carried out in a pin platform box. All components are 3D printed (print files are available on request from the authors). The pin platform (A) contains 96 sockets for securing micromeshes. The lid (B) isolates the pin platform to prevent dehydration. The internal environment is governed by precipitant solution that is secured in 1% agar and is deposited into the moat (C). The window (D) is used to view specimens, to add components through apertures in the lid (E) and to remove excess mother liquor from the micromesh *via* apertures in the bottom of the pin platform box (Fig. 6 ▶). The insets show a close-up view of the MiTeGen micromeshes above the window (including pink precipitant) and a magnified view of the *in situ* crystals. The distance between the agar in the moat and the meshes is 4 mm. The vapor-diffusion setup is equivalent to a sitting drop when the pin platform box is right side up; the box is inverted to achieve a hanging-drop configuration.

Our approach relies on a custom-designed destination plate called a ‘pin platform box’ (Fig. 2 ▶). This box allows the co-crystallization of proteins, precipitants and fragments directly on X-ray data-collection media such as MiTeGen MicroMeshes. Crystal growth is driven by vapor diffusion against a precipitant that is secured in a 1% agar matrix deposited into a long ‘moat’ in the vicinity of the micromesh; placing the agar/precipitant mixture is fast (∼15 min per tray) and easy to do. The crystals can be seen through a ‘window’ of transparent material on both sides of the micromesh that allows components such as purified protein, precipitant and chemicals (including fragments and cryoprotectants) to be added to the micromesh either before or after crystal formation. The window is also transparent to X-rays, so that crystals can be tested for diffraction properties while still in the box (the entire assembly has a standard ANSI/SBS footprint and can be handled by robotic plate scanners). In some cases, the agar and precipitant were added to the window (instead of the moat) to speed crystal formation by decreasing the distance between the protein and the precipitant. To avoid dehydration, components were usually added to the pin platform box through small apertures in the lid after the box had been sealed and equilibrated against the precipitant. Similarly, excess mother liquor can be removed through the bottom of the micromesh using apertures in the pin platform window. The apertures are covered with conventional tape unless material is being added or removed from the micromeshes. At present, we make apertures by hand using a heated metallic probe (∼30 min per tray), so apertures were not used for proteins that did not easily precipitate (lysozyme and thermolysin).

### 2.1. *In situ* crystallization on micromeshes secured in pin platform boxes   

To demonstrate the capability of the Echo 550 to co-crystallize a protein of interest and a fragment library *in situ* directly on data-collection media, we first determined conventional hanging-drop crystallization conditions for three standard protein samples (lysozyme, thermolysin and trypsin) and for a metalloprotein of interest (stachydrine demethylase; Table 1 ▶). For each protein, the same precipitants that were identified in the hanging-drop experiments were loaded into the moat (for trypsin and stachydrine demethylase, in the window) of the pin platform (held in place by a 1% agar matrix). Each pin platform was prepared by maintaining a 2% agar solution at 100°C until the agar transitioned into random coil, and then combining the agar with equal parts of double-concentration precipitant^1^. The agar and precipitant solution was then deposited into the moat (or window) of the pin platform and allowed to solidify. The same conditions that yielded crystals in a conventional hanging-drop experiment were used to yield *in situ* crystals on X-ray data-collection media mounted in a pin platform box. The pin platform box that was used for *in situ* crystallization of lysozyme was re-used on different days; to avoid dehydration, the agar moat was soaked in liquid precipitant solution until needed. For each protein, up to 96 pin-mounted MiTeGen MicroMeshes were snapped into the sockets of a pin platform, and then manually adjusted until each micromesh was in the center of the window. 

A plate-specific definition that allows the *Echo Array Maker* software (Labcyte Inc., Sunnyvale, California, USA) to operate the Echo 550 and dispense liquids to 30 locations on each of the 96 micromeshes is available on request from the authors. This plate definition was made in two stages using *Echo Array Maker*. Firstly, an array definition was generated to access each of the 96 micromeshes on the pin platform. Then, to allow sufficient granularity for the destination locations, the software was used to further partition each micromesh into ten rows by three columns of accessible destination locations using 100 µm grid spacing. Initially, each transfer template was tested for accuracy using water droplets, but this procedure was suspended after gaining confidence in the instrumentation.

After the pin platform had been loaded with agar and precipitant, it was sealed by putting on the lid (Fig. 2 ▶). Each pin platform box assembly was inspected to ensure a snug fit and a hermetic seal. In most cases, experimental components (purified protein, precipitant and fragment) were added to each micromesh through small (∼2 mm) apertures in the lid. Apertures were manually fashioned on the lids using a heated metallic probe with a 1 mm diameter (the lids with apertures can be re-used for multiple co-crystallization experiments). To quickly make apertures, we are developing a robotic hole-puncher in collaboration with a research group that specializes in automation. When not in use, the apertures were covered with a thin strip of adhesive tape (the pin platform box is airtight with the adhesive tape in place). Lysozyme and thermolysin crystals were resistant to desiccation and these fragment screens were concluded before we had perfected these aperture transfer techniques. Cryoprotectant was integrated with the precipitant solution for lysozyme, trypsin and stachydrine demethylase. Thermolysin crystals were cryoprotected by adding 20% ethylene glycol through apertures after the crystals had grown.

Dehydration was virtually eliminated by sealing the source and destination plates, and transferring all liquids through small apertures in the seal. In this study, trypsin and stachydrine demethylase were grown *in situ* with strict adherence to transferring all components through apertures, so that complete control of the environment was maintained at all times. In contrast, aperture transfers were not used in the *in situ* crystallization of lysozyme and thermolysin. Because the use of apertures resulted in marked improvement in the reproducibility and the quality of the crystals (see §[Sec sec3.1]3.1), we conducted a side-by-side comparison of otherwise identically prepared trypsin specimens in which components were transferred through apertures in one case but not in the other case (see §[Sec sec3.4]3.4). In addition to the apertures in the top of the lid, apertures were also fashioned in the bottom of the pin platform windows. These bottom apertures were used to wick away excess mother liquor (when needed) using dental points that were manually pushed upwards until they contacted the bottom of the micromeshes (see §[Sec sec3.5]3.5).

Purified proteins, precipitants and fragments were contained in one or more acoustically transparent 384-well polypropylene source microplates (Labcyte Inc., Sunnyvale, California, USA). In cases where dehydration was not a significant factor (lysozyme and thermolysin), components were added to each micromesh before the pin platform was sealed with its lid. The best results were obtained when specimens were prepared entirely through apertures because this precludes any concentration changes owing to dehydration (trypsin and stachydrine demethylase) or hygroscopic swelling (DMSO compounds). For example, thermolysin was solvated in DMSO, which induced rapid hydration after the liquid was transferred onto the micromesh. DMSO-solvated fragments are expected to induce similar behavior.

Once the specimens were ready for X-ray data collection, the lid was removed from the pin platform box and each micromesh was manually inserted into a MiTeGen Reusable Magnetic Cap and cryocooled by plunging into liquid nitrogen (the reusable caps clamp the pins using a compression fitting, with no adhesive necessary). We are developing a robotic system to automate this step in collaboration with the aforementioned automation research group.

### 2.2. *In situ* co-crystallization of proteins with fragment libraries   

To demonstrate the capability of the Echo 550 to identify novel ligands by co-crystallizing proteins with fragment libraries *in situ* directly on data-collection media, we prepared a ‘mini-library’ of compounds (solvated in water, DMSO or ethanol) and co-crystallized these compounds with three commercial test proteins (lysozyme, thermolysin and trypsin) and with one expressed protein (stachydrine demethylase), as described in §[Sec sec2.1]2.1. Some of the compounds were previously reported ligands (*N*-acetylglucosamine, aspartic acid, benzamidine and l-proline), and others were unknowns. In all, our fragment mini-library (including known ligands) consisted of 33 compounds (four in DMSO and one in 25% ethanol; Supplementary Table S1^2^). The compounds in our mini-library were chosen largely because of safety guidelines, since our workforce for this study was predominantly undergraduate students in short-term appointments (spring and summer terms of 2013). The mean mass of our mini-library (159 Da) was similar to published best practices (268 Da) (values obtained from *ChemSpider*; Pence & Williams, 2010 ▶). However, the mean lipophilicity of our mini-library (−2.08) was lower than published best practices (2.10; Keserü & Makara, 2009 ▶). Each protein was checked for DMSO compatibility in two ways before beginning the study. Crystal appearance and/or diffraction quality began to decay at a DMSO concentration of 5% for lysozyme, 40% for thermolysin, 20% for trypsin and 10% for stachydrine demethylase. In a typical experiment, 2.5 nl of each fragment was added to 10 nl of protein and precipitant. In the case of thermolysin, smaller volumes were used (2.5 nl of protein and precipitant). A freely accessible stop-motion video of a 2.5 nl drop of thermolysin (330 mg ml^−1^ thermolysin in 50 m*M* Tris pH 7.5, 45% DMSO, 1.4 *M* CaCl_2_) co-crystallized *in situ* with 2.5 nl l-­asparagine (300 m*M*) directly on a micromesh can be viewed at http://www.youtube.com/channel/UCtCiMjlzBnq5VYZzrEi3EiQ/videos. Crystallization conditions for all four proteins are shown in Table 1 ▶. Note that in all cases the same conditions that produced crystals on a conventional cover slip also produced crystals directly on micromeshes.

Every co-crystallization micromesh (containing protein, precipitant and fragment) was examined using a Leica microscope. The micromeshes that contained crystals were cryocooled by plunging into liquid nitrogen as described in §[Sec sec2.1]2.1. Diffraction data were collected on beamlines X12C, X25 and X29 at the National Synchrotron Light Source (NSLS). Data sets were processed with *HKL*-2000 (Otwinowski *et al.*, 2001 ▶) and further processed using *CTRUNCATE* in the *CCP*4 suite (Winn *et al.*, 2011 ▶). Structures were obtained by molecular substitution from published models and refined using *REFMAC* (Murshudov *et al.*, 2011 ▶) and *ARP*/*wARP* [Perrakis *et al.*, 2001 ▶; the starting models were PDB entries 1lyz for lysozyme (Diamond, 1974 ▶), 4tln for thermolysin (Holmes & Matthews, 1981 ▶), 4i8g for trypsin (Liebschner *et al.*, 2013 ▶) and 3vca for stachydrine demethylase (Daughtry *et al.*, 2012 ▶)]. Binding fragments were identified in an *F*_o_ − *F*_c_ difference map by visual inspection. A computer algorithm was used to confirm the binding and to compute the best occupancy (*PHENIX*; Adams *et al.*, 2010 ▶). We then used *AutoDock Vina* (Trott & Olson, 2010 ▶) to compare each fragment that was observed to bind in the co-crystallization experiments with the best predicted pose for the same fragment (see Supporting Information).

### 2.3. Multiple *in situ* protein plus fragment assays on one data-­collection support   

The reported techniques can rapidly prepare specimens for X-­ray-based fragment hit discovery projects, and modern synchrotrons can generate a complete data set in under 1 s (Hodgson *et al.*, 2009 ▶). Conversely, the duty cycle of existing cryogenic automounters limits the maximum achievable throughput to about one screened structure per minute. One approach to circumvent this speed limit is to position multiple discrete fragment co-crystallization experiments onto each data-collection micromesh. To this end, MiTeGen collaborated with our group to design high-density micromeshes with a capacity to accomodate as many as ten discrete experiments (Fig. 4). To test these high-density formats, we used the high ‘drop-on-drop’ positional precision of the Echo 550 to co-crystallize 10 nl thermolysin (as described in §[Sec sec2.2]2.2) combined with 10 nl 100 m*M* l-histidine and, separately, with 10 nl 100 m*M* l-asparagine. A fragment-free control was also positioned on the same micromesh. All three co-crystals yielded interpretable diffraction. A freely accessible stop-motion video tracks these crystals as they grow in the three conditions, and can be viewed at the URL given in §[Sec sec2.2]2.2.

## 3. Results   

All of the data reported in this study were collected from co-­crystallization experiments with the fragment mini-library. However, it is also possible to grow native crystals *in situ* on data-collection micromeshes and then acoustically combine the already grown crystals with the fragment library. To explore this possibility, we compared the results obtained by *in situ* co-crystallization of thermolysin with a fragment mini-library with the results obtained when native *in situ* crystals were soaked overnight in the presence of the same fragments (data not shown). Although the results were broadly similar, in one case the same fragment was observed to bind in a different conformation and location depending on the technique used. Soaking and co-crystallization strategies are complementary, and often yield different false negatives. Soaking may overlook binding sites that are occluded by interprotein contacts, and co-crystallization may disturb the chemical environment and prevent crystal growth. Consequently, users of the *in situ* co-crystallization technique are advised to be aware that the binding properties of fragment hits discovered through co-crystallization may differ from the binding properties of fragment hits discovered through soaking experiments.

### 3.1. *In situ* crystallization conditions are similar to hanging drops   

In a conventional crystallization experiment, when the working volume falls below a few hundred nanolitres differences often arise in the composition and concentration of precipitants that are needed to induce crystallization. We did not observe this trend when comparing our hanging-drop and *in situ* crystallization experiments for test crystals (Table 1 ▶). We believe the underlying cause of the reported variations between crystallization conditions at nanolitre *versus* microlitre volumes is largely because very small droplets are susceptible to dehydration (with some liquid-handling methods, the uncertainty in the volume transferred will also increase with small volumes). Using *in situ* crystallization, this variation can be prevented by strict adherence to transferring all liquids through an aperture (from a sealed 384-well polypropylene source microplate and into a sealed pin platform box which has reached equilibrium with its precipitant solution). Table 1 ▶ illustrates that careful avoidance of dehydration (by monitoring susceptibility and using aperture transfers when necessary) yields identical crystallization conditions for conventional hanging-drop experiments compared with acoustically prepared *in situ* experiments.

### 3.2. *In situ* co-crystallization experiments yield high-quality data and identify fragment hits   

The reservoir of the pin platform box is a shared precipitant that drives the crystallization of up to 96 protein plus fragment trials in the box. This design accelerates specimen preparation, but precludes tuning the precipitant mixture for each fragment. For each test protein, a binary yes/no summary for chemicals that were compatible with the shared crystallization solution is shown in Supplementary Table S1 (crystallization is denoted by an X; binding is denoted by the occupancy). Our results are encouraging because despite using very high concentrations of fragments (usually 100 m*M*; see Supplementary Table S1), most of the co-crystallization trials yielded high-quality crystals that generated good diffraction data and interpretable electron density. This supports the feasibility of using a shared reservoir to unambiguously determine a yes/no answer for the success of most co-crystallization trials.

**Figure 3:**
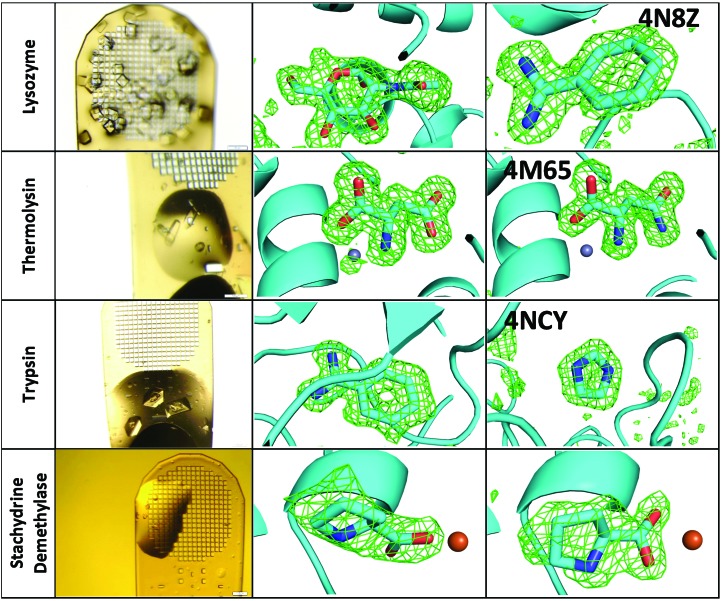
*In situ* crystallization and fragment hit discovery directly on micromeshes. The pictures on the left show crystals grown *in situ* on the micromeshes. The pictures in the middle show electron density around known ligands (OMIT difference contoured at 3σ) that were co-crystallized with protein (*N*-­acetylglucosamine with lysozyme, l-aspartic acid with thermolysin, benzamidine with trypsin and l-proline with stachydrine demethylase). The pictures on the right show electron density around previously unreported fragment hits that were discovered using *in situ* co-crystallization (benzamidine with lysozyme, l-asparagine with thermolysin and imidazole with trypsin) and one unreported fragment (cleaved *N*-methylproline) that was previously discovered by one of the authors (see §[Sec sec3.2]3.2). Note that the methyl moiety of *N*-methylproline is immediately cleaved in response to X-ray photoactivation of the enzyme (because of this, the methyl moiety is not shown in the figure). PDB codes are indicated for deposited structures.

For each of the proteins examined, co-crystallization trials against our mini-library revealed one previously unreported fragment hit. All of these hits were confirmed by conventional hanging-drop follow-up experiments. For lysozyme and thermolysin, the hanging-drop experiments yielded larger and better diffracting crystals, and these data were deposited in the PDB. In the case of trypsin, the original screening crystal yielded the best data. The stachydrine demethylase data are being deposited in combination with a separate manuscript. The known ligand was also confirmed in each protein. Fig. 3 ▶ summarizes the outcome for the highest occupancy binding observed in each case.

#### 3.2.1. Lysozyme   

Lysozyme crystallization was resistant to desiccation, so protein, precipitant and fragments were added to an open pin platform box and the cover was fitted after all components had been added. *In situ* lysozyme crystals formed in thin layers that were suitable for data collection without removing excess mother liquor. Three of the compounds tested in our mini-library were incompatible with the crystallization procedure. The known *N*-acetylglucosamine ligand was easily identified in the electron density. Benzamidine was also observed to bind to lysozyme, a previously unreported result (PDB entry 4n8z). The top scoring pose in a simulation using *AutoDock Vina* correctly positioned benzamidine in the same location and orientation as observed in the electron density (0.21 Å average coordinate error; see Supplementary Table S2).

#### 3.2.2. Thermolysin   

Once thermolysin crystals were observed on the micromeshes, cryoprotectants were added to each crystal. The thermolysin crystallization solution is highly hygroscopic. Consequently, the aggregate volume on these micromeshes was excessive, which caused unacceptable background in the X-ray diffraction. To address this problem, each thermolysin micromesh was dab-dried using a ‘dental point’ (Gutta Percha #13985) through an aperture at the bottom of the pin platform window. Using X-ray data, binding was observed both for the known ligand (aspartic acid) and for a previously unreported test fragment (asparagine; PDB entry 4m65). The binding location and binding geometry observed using the X-ray data were the same for aspartic acid and for asparagine. However, the best predicted binding pose (the top-scoring *AutoDock Vina* pose) had a slightly different binding geometry (1.15 Å coordinate error; see Supplementary Table S2).

#### 3.2.3. Trypsin   

Because trypsin crystallization was very sensitive to desiccation, all transfers of protein, precipitant and fragments were carried out through apertures in the pin platform box cover. As a consequence, the results obtained from trypsin crystals were markedly more reproducible than the results obtained from lysozyme and thermolysin crystals. The overall quality of the trypsin crystals, as well as the observed X-ray diffraction patterns, was also superior. The crystals diffracted to 1.1 Å resolution and consequently no follow-up experiment was needed to produce publication-quality data for benzamidine (a known control) or imidazole (a previously unreported allosteric binding mode that is distant from the active site; PDB entry 4ncy). Imidazole binding caused significant conformational changes in the surrounding region of the protein (Supplementary Fig. S2). To demonstrate the utility of *in situ* fragment screening, all 33 X-­ray data sets from the trypsin co-crystallization with our mini-library were used to generate refined structures (see §[Sec sec2.2]2.2). Data from this first-pass co-crystallization trial are shown in Supplementary Fig. S1). A picture of the crystal and a rendition of the resulting electron density (difference OMIT map contoured at 3σ) are shown for (i) the known benzamidine ligand in the best diffracting no-hit screen, (ii) the known benzamidine ligand in the worst diffracting no-hit screen and (iii) the previously unreported imidazole ligand. The two top-scoring *AutoDock Vina* poses reproduced the observed binding location (0.78 Å average coordinate error), but the rings were rotated by 72° to accommodate two binding geometries with equal energies (see Supplementary Table S2). Trypsin was one of the targets of the SAMPL challenge for identifying ligand hits to support modelling work (Newman *et al.*, 2009 ▶), so a very large number of trypsin ligands are reported in the PDB. Our follow-up experiments reproduced the two previously reported imidazole binding locations (PDB entries 3qk1 and 1y59; Schopfel *et al.*, 2011 ▶; Di Fenza *et al.*, 2007 ▶).

#### 3.2.4. Stachydrine demethylase   

Initially, *in situ* crystallization of stachydrine demethylase was a challenge. After multiple attempts at crystallization, it was clear that desiccation was driving irreproducibility because the crystallization results were sensitive to the order in which the specimens were prepared. The first specimens to be prepared failed to crystallize because they dehydrated during the time needed to prepare the later specimens. To overcome the stubborn irreproducibility, the experiments had to be repeated with strict adherence to transferring all specimens through apertures. Once this had been performed, we were able to reproduce results previously demonstrated by our group (Agarwal *et al.*, 2014 ▶). When l-proline was co-crystallized with stachydrine demethylase, the l-proline was observed in the electron density. In contrast, when *N*-­methylproline was co-crystallized with stachydrine demethylase, only l-proline was observed in the electron density (electron density was not observed for the methyl moiety). This supports the hypothesis that exposure to X-rays quickly induces enzymatic cleavage of the *N*-methyl group, leaving behind l-proline.

### 3.3. One protein co-crystallized against three discrete fragments on one high-density micromesh confirms multiple fragment hits   

**Figure 4:**
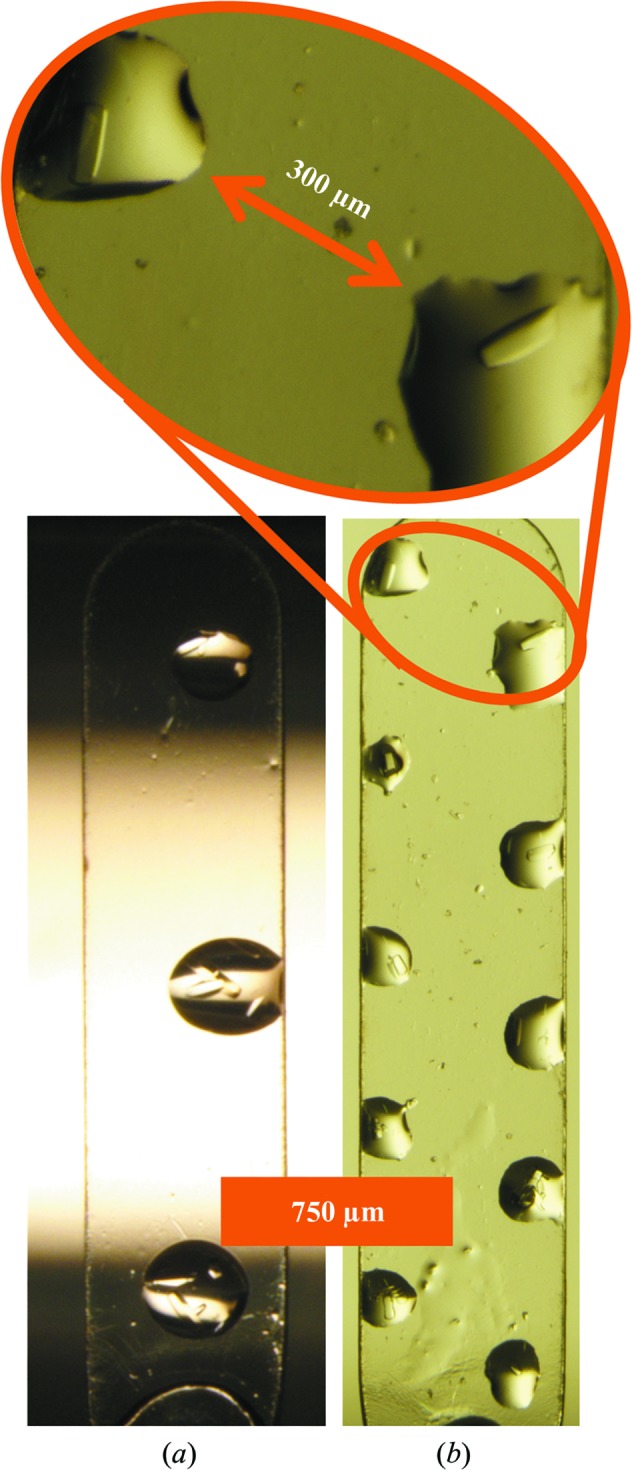
Placing multiple experiments on a single specimen holder allows the goniometer to act as an ‘auxiliary robotic automounter’ that rapidly translates between experiments during the time that the conventional cryogenic automounter completes its duty cycle. (*a*) shows *in situ* co-crystallization of thermolysin, precipitant, cryoprotectant and fragments. In this example the fragments were asparagine (bottom), histidine (middle) and a control with no ligand (top). The custom specimen holder has sufficient space to accommodate ten discrete experiments (*b*) (with no ligands or cryoprotectants). Even with such a high density of specimens, the spacing between droplets is adequate to prevent cross-contamination (inset). A video is available on our website showing multiple crystals growing *in situ* directly on custom MiTeGen paddles, each with a different fragment (URL in §2.2).

To fully utilize the available brightness of modern synchrotron sources, specimens will have to be delivered to the X-ray beam much faster than the duty cycle of cryogenic automounters. Although the majority of the data for this study were generated using a traditional ‘one experiment per micromesh’ concept, we also tested the feasibility of using high-density specimen supports so that data-acquisition rates could exceed the duty cycle of the mounting system. We demonstrated that three distinct protein plus fragment co-­crystallization trials could be co-positioned on a single micromesh. Moreover, the physical space on the high-density micromesh is adequate to support up to ten experiments of this type (Fig. 4 ▶). The implied sustained throughput is 600 fragments screened per hour. This throughput approaches the maximum data-acquisition rate that can be supported by the available X-ray brightness of best-in-class third-generation synchrotrons using a 20 × 20 µm beam size.

### 3.4. Transferring components through apertures to prevent desiccation   

**Figure 5:**
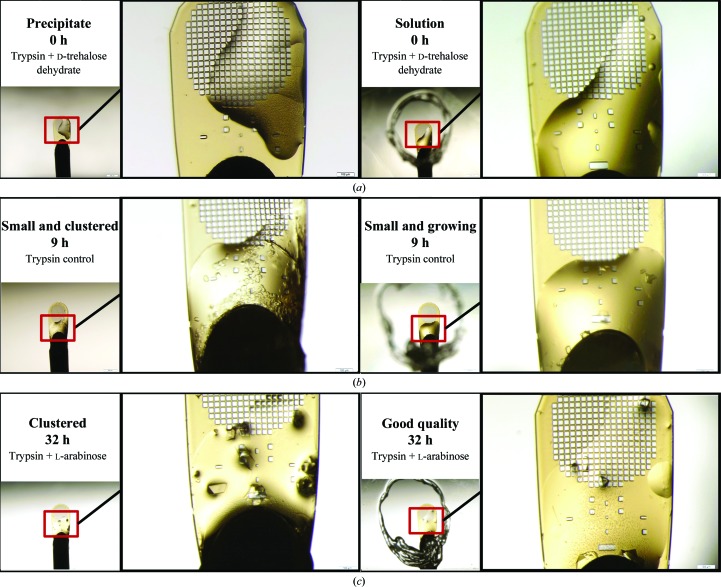
Side-by-side comparison of trypsin *in situ* co-crystallization, with ejections made with and without apertures in separate experiments that simulate the screening of 96 different fragments. Each panel shows a close-up view of a micromesh containing the same protein, precipitant and fragment. Micromeshes prepared without the use of apertures are shown on the left and micromeshes prepared with the use of apertures are shown on the right. Dehydration, precipitation and low-quality crystal formation were observed when apertures were not used (examples of each failure mode are shown at different stages of the crystallization process). In contrast, the samples that were prepared using apertures did not show dehydration damage. The samples that precipitated immediately after the ejections (*a*, left) did not improve with time. Three samples are shown: (*a*) protein, precipitant and d-­trehalose, (*b*) protein and precipitant control and (*c*) protein, precipitant and l-arabinose. In all three cases, the defining characteristic of successful co-crystallization for these trypsin screens was rigorous control of the environment to prevent desiccation.

Many groups have observed instability in the composition and concentration of the precipitants needed as crystallization volumes fall below 100 nl. This may be caused by desiccation in non-microfluidic micro-crystallization techniques (Totir *et al.*, 2012 ▶). Exposure to the uncontrolled laboratory atmosphere is expected to impact small volumes (in the few nanolitres range) much more rapidly than larger volumes (in the few microlitres range). To test this hypothesis, we simulated a side-by-side comparison of 96 co-crystallization trials with and without the use of apertures. The simulation consisted of ten *in situ* co-crystallization trials (each with one fragment screen). Each co-crystallization trial was separated by ten two-component transfers to a dummy location, to simulate the total time that would be required for a genuine 96-well preparation (Fig. 5 ▶). Our results demonstrate that preventing desiccation by transferring all components through apertures is the defining factor that determines the success of the experimental protocol. When trypsin specimens were prepared using apertures, the experiment was successful. However, when apertures were not used, the protein either precipitated or formed small low-quality crystal clusters. Improvements in crystal growth were also observed when stachydrine demethylase co-crystallization was performed using apertures.

### 3.5. Removing excess mother liquor to improve the signal-to-noise ratio   

**Figure 6:**
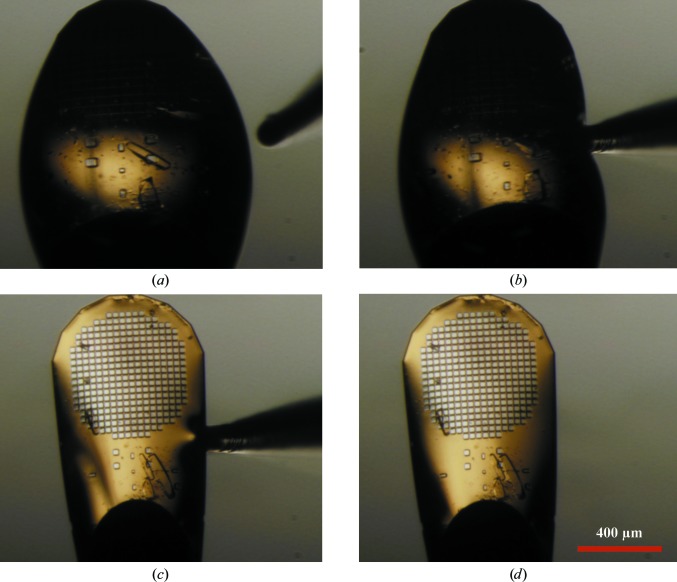
Dab-drying thermolysin crystals that were co-crystallized *in situ*. The thermolysin crystals grew well, but were enveloped by excess mother liquor consisting of buffer solution, precipitant solution, fragment solution and water that was incorporated owing to the hygroscopic nature of DMSO (DMSO is present in both the thermolysin crystallization condition and the solvated fragments). For demonstration purposes, we prepared a specimen with a hugely exaggerated volume of water-engorged DMSO. To improve the signal to noise, each crystal-containing micromesh was individually dab-dried by thrusting an absorbent dental point vertically upwards through an aperture in the bottom of the pin platform box window (*a*) (the vertical absorbent point appears inclined owing to angular optics in the dissecting microscope). The absorbent point then touched the bottom of the micromesh (*b*) and excess mother liquor was steadily withdrawn through the holes in the micromesh (*c*), leaving the crystals surrounded by a thin blanket of mother liquor (*d*). Crystals that had excess mother liquor withdrawn in this way were compared with untreated crystals. The diffraction limit at unity *I*/σ(*I*) was observed to be 1.9 Å for dab-dried crystals *versus* 3.7 Å for untreated crystals (data not shown).

*In situ* crystallization of thermolysin resulted in well diffracting crystals surrounded by a swollen envelope of mother liquor. The excess mother liquor consisted of protein solution, precipitant solution, fragment solution and water that was incorporated owing to the hygroscopic properties of DMSO. To improve the signal to noise, excess mother liquor was removed by thrusting dental points upwards through an aperture in the bottom of the window and vertically up to the bottom of each mesh. The absorbent points then steadily removed the excess mother liquor through the micromesh grid, leaving the protein crystals unharmed and surrounded by a thin blanket of mother liquor. This procedure was highly effective (Fig. 6 ▶), and in hindsight could also have benefited the quality of the other three *in situ* crystallization efforts.

## 4. Discussion   

Fragment screening using X-ray crystallography immediately links the fragment hit discovery step to powerful structural tools such as competence for allosteric detection, binding-mode determination and insights for improving the characteristics of the candidate drug, such as pharmacokinetics (Blundell & Patel, 2004 ▶). Supplementary Fig. S2 illustrates a significant conformational adaptation of trypsin in response to allosteric binding of an imidazole fragment. This highlights the promise of *in situ* co-crystallization to uncover novel therapeutic compounds that modify the structure and/or function of health-related macromolecules.

One major limitation of using X-ray crystallography as the primary screening tool is that data-collection rates impose an upper limit to the attainable screening speed. However, the very high brilliance of third-generation synchrotrons can mitigate this limit with the assistance of suitable technologies for rapidly preparing and delivering specimens to the X-ray beam. Acoustic droplet ejection is a robust technology for rapidly preparing protein crystals and for rapidly delivering protein crystals to the X-ray beam, either on micromeshes (Soares *et al.*, 2011 ▶) or on a moving conveyor belt (Roessler *et al.*, 2013 ▶).

We have demonstrated that acoustic methods enable fragment screening using very low volumes of protein and fragment (2.5 nl) at a high speed of specimen preparation (60 specimens per minute) and with rapid data acquisition (ten data sets per minute). This acoustic strategy for *in situ* co-crystallization directly on data-collection media is remote-compatible and can potentially deposit machine-generated metadata directly into a universal database. The high throughput enables screening highly concentrated (∼100 m*M*) fragments one at a time, and avoids chemical cocktails and inter-fragment reactivity (Hubbard, 2008 ▶) and the danger that a high aggregate fragment concentration may interfere in protein or crystal integrity (Shrake & Ross, 1990 ▶; Chi *et al.*, 2003 ▶). We identified a total of three unreported fragment hits, one for each test protein, by screening against a 33-component fragment mini-library. The average occupancy was 80%. These data suggest that acoustic *in situ* co-crystallization of proteins and fragments directly on micromeshes is a vigorous and effective strategy for lead discovery and drug development.

Surface plasmon resonance (SPR) is a conventional strategy for analysis of interactions with and between macromolecules used for active-site independent screening of fragment libraries in drug discovery, where proteins are tethered to a gold chip (Myszka & Rich, 2000 ▶). Along with X-ray crystallo­graphy, SPR can identify allosteric compounds (Vanderpool *et al.*, 2009 ▶), and the two methods yield complementary information about fragment hits (kinetics from SPR, binding orientation from crystallography). SPR search strategies involve careful pre-preparation of each gold chip, individually tailored to each protein, to perfect the protein-tethering strategy and the pilot screen controls (Löfås & McWhirter, 2006 ▶); this process typically depletes 5 µg from the supply of purified protein (Giannetti, 2011 ▶; Rich & Myszka, 2004 ▶). A systematic analysis for the SAMPL challenge required about one week to screen 384 samples, including data analysis (Newman *et al.*, 2012 ▶). Higher throughput rates are achieved using multiple gold chips or using pintool spotting (Neumann *et al.*, 2007 ▶). Specimen consumption is estimated as 100 ng of purified protein and 25 µg of fragment chemicals per screened condition. Sensitivity is high (1 m*M*; Myszka & Rich, 2000 ▶); however, both false positives and false negatives are difficult to detect without time-consuming confirmation experiments (Giannetti, 2011 ▶). SPR and the fast compact serial crystallography described here are synergistic methods for fragment screening, both because they yield complementary information (kinetics and pose) and because each method can identify false positives generated by the other.

Regulatory therapeutic compounds often interact with the target protein away from the active site (Sijbesma & Nolte, 1991 ▶). Hence, a remaining challenge for fragment-based drug discovery is a strong strategy for identifying allosteric pharmaceuticals to treat nonpathogenic diseases such as genetic, degenerative and cognitive deficit disorders (Harms *et al.*, 2013 ▶). Structure-based drug discovery is competent to identify such alternative binding sites; however, it has been limited by challenging specimen preparation, sluggish data acquisition, a difficult to automate crystal-mounting step and high consumption of both purified protein and chemicals. To overcome these challenges, we have developed a fragment-screening method using *in situ* co-crystallization of proteins and fragments directly on data-collection micromeshes. The process is extraordinarily parsimonious for purified protein (2.5–25 nl per screen) and chemical libraries (2.5 nl per fragment). The speed of specimen preparation (60 per minute) and data acquisition (ten per minute) can keep up with the high flux of third-generation synchrotrons. The Echo 550 automatically generates metadata, and this information can be made available to the data-acquisition software. Finally, since the crystals are grown *in situ*, we have eliminated the specimen-mounting step, along with possible cross-contamination, crystal losses, dehydration and mechanical damage to the crystals, all of which may occur during mounting. One key remaining challenge, currently under development by our collaborators, is the design of a simple robotic solution for cryo-plunging pins and inserting them into cryogenic pucks.

Any sober evaluation of the potential offered by novel drug-discovery processes must consider that promising answers to this challenge have frequently encountered unexpected roadblocks. One frequent unwelcome guest has been the cryptic false positive, which is revealed by extensive additional investigation. This problem is somewhat mitigated by structure-based methods, since inspection of the electron-density map usually differentiates clear binders from marginal cases. Other techniques are highly sensitive to small errors in concentration, incorrect setup, inadequate controls or are limited to certain classes of proteins (for example, some methods are incompatible with membrane proteins or very large proteins). In contrast, *in situ* structure-based methods are robust enough that the majority of the specimen preparation was carried out by undergraduate students during a ten-week summer internship. This new technology promises hope for identifying allosteric drugs to treat disorders that have been inaccessible to structure-based drug discovery owing to low protein expression, constraints to fragment-search strategy or insufficient throughput speed.

## Supplementary Material

PDB reference: thermolysin, 4m65

PDB reference: trypsin, 4ncy

PDB reference: lysozyme, 4n8z

Supporting Information.. DOI: 10.1107/S1399004713034603/nj5173sup1.pdf

## Figures and Tables

**Table 1:** Crystallization, data-collection and model-refinement statistics

	Lysozyme	Thermolysin	Trypsin	Stachydrine demethylase
Crystallization conditions				
Strategy				
Hanging drop				
Protein (µl)	4	2	4	2
Precipitant (µl)	4	2	4	2
Fragment (µl)	2	1	2	1
*In situ*				
Protein (nl)	20	1.25	20	5
Precipitant (nl)	20	1.25	20	5
Fragment (nl)	10	2.5	10	2.5
Protein				
Hanging drop	120 mg ml^−1^	330 mg ml^−1^ + 45% DMSO	30 mg ml^−1^ + 10 mg ml^−1^ benzamidine	10 mg ml^−1^ + 25 m*M* hexaamine CoCl_2_
*In situ*	120 mg ml^−1^	330 mg ml^−1^ + 45% DMSO	30 mg ml^−1^ + 10 mg ml^−1^ benzamidine	10 mg ml^−1^ + 25 m*M* hexaamine CoCl_2_
Buffer				
Hanging drop	0.1 *M* sodium acetate pH 4.6	50 m*M* Tris pH 7.5	10 m*M* CaCl_2_ + 20 m*M* HEPES pH 7	0.1 M HEPES pH 7
*In situ*	0.1 *M* sodium acetate pH 4.6	50 m*M* Tris pH 7.5	10 m*M* CaCl_2_ + 20 m*M* HEPES pH 7	0.1 *M* HEPES pH 7
Precipitant				
Hanging drop	4% NaCl	1.45 *M* CaCl_2_	20% PEG 8000 + 200 m*M* ammonium sulfate + 100 m*M* bis-tris	7.5% PEG 3350 + 10% glycerol
*In situ*	4% NaCl	1.45 *M* CaCl_2_	20% PEG 8000 + 200 m*M * ammonium sulfate + 100 m*M* bis-tris	7.5% PEG 3350 + 10% glycerol
Data-collection statistics				
X-ray source				
Screening	NSLS X29	NSLS X25	NSLS X25	NSLS X25
Confirmation	NSLS X12C	NSLS X12C	NSLS X12C	NSLS X25
Wavelength (Å)	1.1	1.1	1.1	1.1
Beam size (µm)	75 × 75	75 × 75	50 × 50	50 × 50
Resolution (Å)	1.6 (39.2)	1.6 (31.6)	1.4 (28.3)	2.3 (41.9)
*R*_merge_ (%)	11.4 (94.8)	4.8 (43.2)	3.6 (15.4)	7.9 (57.7)
〈*I*/σ(*I*)〉	14.9 (76.0)	58.42 (6.32)	46.31 (10.25)	26.5 (0.8)
Completeness (%)	99.2 (91.4)	98.1 (96.6)	99.7 (94.4)	91.6 (52.1)
Multiplicity	22.8	28.2	5.9	18.2
Model-refinement statistics				
No. of reflections	14409	43035	38155	21941
*R*_work_/*R*_free_ (%)	17.29/20.89	13.0/15.9	11.95/14.31	21.37/26.70
R.m.s. deviations				
Bond lengths (Å)	0.022	0.029	0.024	0.016
Bond angles (°)	2.096	2.559	2.644	1.784

## Footnotes

1 Some precipitants may inhibit agarose-gel formation (for example, >2 *M* salts and >50% PEGs). In these cases, the agarose gel is prepared without precipitant and the precipitant is soaked into the gel afterwards. We successfully used this solution with photosystem II crystals (the PS2 mother liquor contains 40% PEG 5000 and agarose will not harden in its presence).

2 Supporting information has been deposited in the IUCr electronic archive (Reference: NJ5173).
