# Supplementary material for: Hitting the target: fragment screening with acoustic in situ co-crystallization of proteins plus fragment libraries on pin-mounted data-collection micromeshes
Source: Acta Crystallogr D Biol Crystallogr. 2014 Apr 30;70(Pt 5):1177–89. doi: 10.1107/S1399004713034603 (PMC4014116; doi:10.1107/S1399004713034603)
Supplement: Supplementary file 1 [file d-70-01177-sup1.pdf]

Supporting Information

Supporting Methods

The potency of each fragment hit in a structure based discovery project is usually improved by means of modification through medicinal chemistry (Rees et al., 2004) or by linking multiple fragments together (Cheng et al., 2011), to generate a lead compound that is suitable for optimization. Computational techniques typically assist this process. The first step is to reproduce the observed fragment binding geometry *in silico*. We used *AutoDock vina* (Trott and Olson, 2010) to compare each fragment that was observed to bind in the co-crystallization experiments with the best predicted pose for the same fragment in that region. Each refined protein atomic model was partitioned into slabs and screened to confirm that the protein bound to the predicted fragments (the slabs were long and wide enough to enclose the entire protein, and as thick as our computational memory resources allowed). For each fragment that was observed to bind, the predicted pose that bound most robustly was recorded and compared to the observed orientation for that fragment.

Supporting Table S1: Co-crystallization trials of lysozyme (“Lys”), thermolysin (“TL”), trypsin (“Tryp”), and stachydrine demethylase (“StDem”) combined with a mini-library of 33 fragments. Possible results were no crystal observed (**Ø**), fragment not observed to bind (**X**) or the calculated occupancy (**occ%**) for each fragment.

**Fragment Name**	**Wt. g/mol**	**Vol. Å^3**	**cLogP**	**Conc. mM**	**Crystallization and binding**			
					**Lys**	**TL**	**Tryp**	**StDem**
ADA[1]	190	161	-3.89	100	**X**	**X**	**Ø**	**X**
ADPA[2]	184	175	2.58	100	**X**	**X**	**Ø**	**X**
Ammonium Acetate	77	54	-0.22	100	**X**	**X**	**X**	**X**
Ammonium Bicarbonate	79	45	0.25	100	**X**	**X**	**X**	**X**
Ammonium Tartrate	184	113	-1.83	100	**X**	**X**	**X**	**X**
L-Arabinose	150	133	-2.94	100	**X**	**Ø**	**X**	**X**
L-Arginine Hydrochlor.	211	163	-3.24	67	**Ø**	**X**	**X**	**X**
L-Ascorbic Acid	176	139	-1.91	100	**X**	**X**	**X**	**X**
L-Asparagine MonoH_2O	132	115	-4.29	100	**X**	**100%**	**X**	**X**
L-Aspartic acid	133	113	-3.50	3	**X**	**100%**	**X**	**X**
Benzamidine	120	115	0.89	100	**100%**	**X**	**100%**	**X**
Bicine	163	154	-4.37	100	**X**	**X**	**X**	**X**
Bis-Tris Propane	282	276	-4.67	100	**X**	**X**	**X**	**Ø**
Glycine	75	68	-3.41	100	**X**	**X**	**X**	**X**
Gly Gly	132	115	-4.52	100	**X**	**X**	**X**	**X**
Gly Gly Gly	189	163	-5.62	100	**X**	**X**	**X**	**X**
Guanidine	59	55	-1.24	100	**X**	**X**	**X**	**X**
L-Histidine	155	135	-3.29	100	**X**	**Ø**	**X**	**X**
8-Hydroxyquinoline	145	129	1.83	2	**X**	**X**	**X**	**X**
Imidazole	68	62	-0.15	100	**X**	**X**	**100%**	**X**
Inosine	268	213	-2.48	67	**X**	**X**	**Ø**	**X**
MES[3]	195	169	-2.49	100	**X**	**X**	**X**	**X**
MOPS[4]	209	186	-2.43	100	**X**	**X**	**X**	**X**
Molybdic Acid	161	79	-1.78	83	**Ø**	**Ø**	**X**	**X**
N-Acetyl-Glucosamine	221	193	-3.22	100	**100%**	**X**	**X**	**Ø**
N-Methyl-Proline	129	125	-2.44	100	**X**	**X**	**X**	**40%**
Pot. Thiocyanate	97	34	-0.54	100	**X**	**X**	**X**	**Ø**
Succinic Acid	118	101	-0.40	100	**X**	**X**	**X**	**Ø**
TAPSO[5]	259	222	-5.11	100	**X**	**X**	**X**	**X**
TetMeth-PhDiamine[6]	164	175	2.19	100	**X**	**X**	**Ø**	**X**
Thymine	126	106	-0.46	100	**X**	**X**	**Ø**	**X**
D-Trehalose Dihydrate	342	289	-4.70	100	**X**	**Ø**	**X**	**X**
Urea	60	53	-1.36	100	**Ø**	**X**	**X**	**X**
**Average**	**159**	**134**	**-2.08**					

[1]N-(2-acetamido)-iminodiacetic acid
[2]4-aminodiphenylamine
[3]2-(N-Morpholino)ethanesulfonic acid
[4]3-(N-Morpholino)propanesulfonic acid
[5]2-Hydroxy-3-[[2-hydroxy-1,1-bis(hydroxymethyl)ethyl]amino]propanesulphonic acid
[6]N,N,N',N'- Tetramethyl-p-phenylenediamine

Supporting Table S2: Binding locations and geometries verified using *AudoDock Vina* (data from the top scoring pose are shown). The binding energy is shown for each top scoring pose, along with the coordinate error (Å) between each pose and the fragment observed in the electron density. In each case, a stick diagram shows the overlay between the binding geometry observed in the x-ray data and the binding geometry predicted by *AutoDock Vina*. In the case of lysozyme with benzamidine, the location and orientation of the top scoring pose were almost identical (0.21Å average coordinate error). The asparagine was observed to bind in the same location as predicted, but with reversed geometry. In the case of trypsin + imidazole, there were two high scoring *AudoDock Vina* poses; both were in the same location as the observed fragment, but rotated to accommodate two binding geometries with equal energies.

Protein	Ligand	Binding energy	Coordinate Error(Å)	Pred. & obs. overlay
Lysozyme	Benzamidine	-5.4	0.21	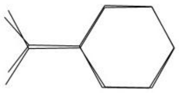
Thermolysin	Asparagine	-4.4	1.15	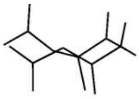
Trypsin	Imidazole	-3.0	0.78	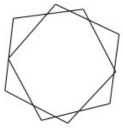

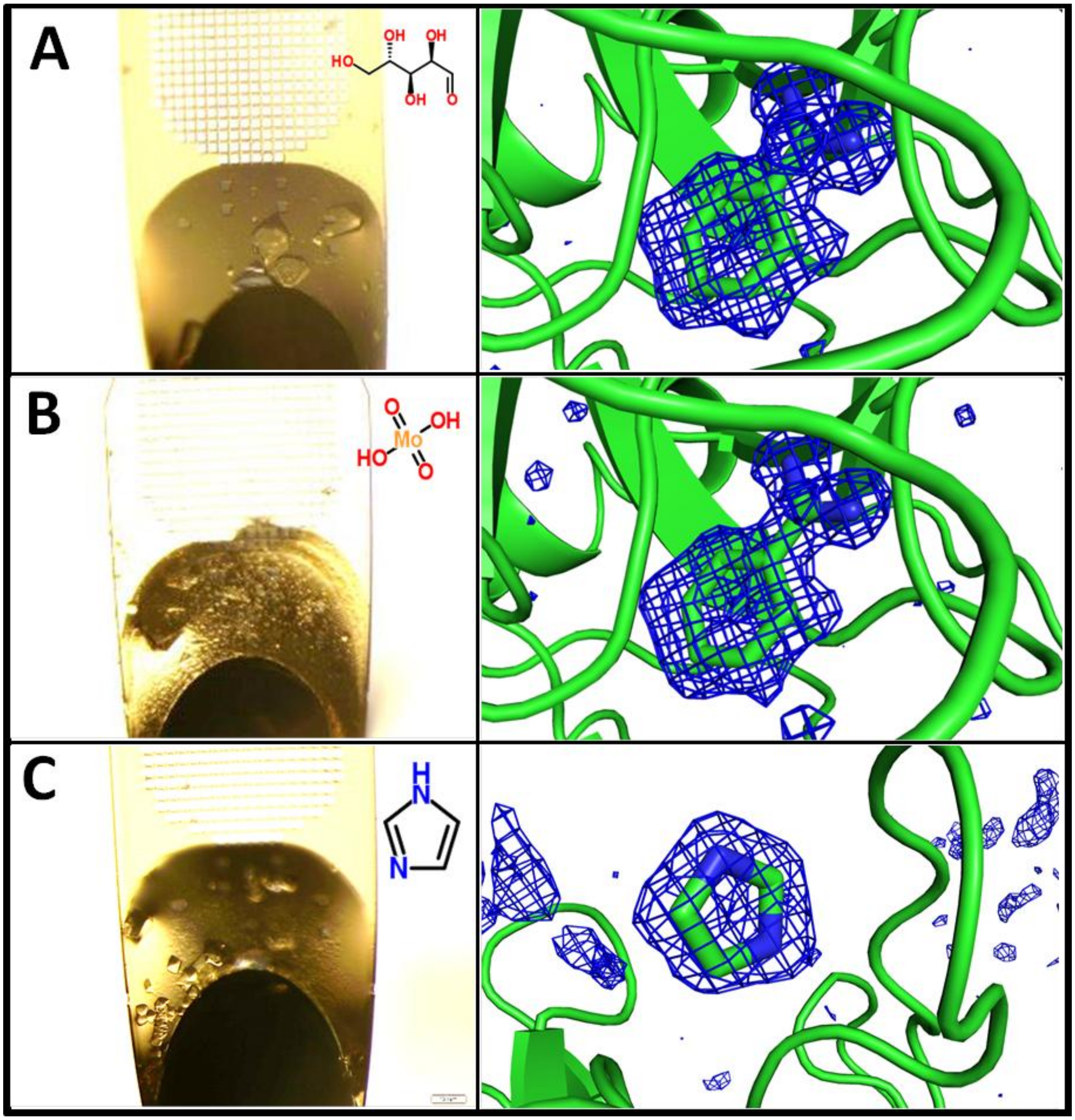

Supporting Figure S1: *In situ* co-crystallization of trypsin. Two examples of non-binding fragments and one fragment hit are shown. In each case, the fragment structure is inset in the micromesh / crystal panel. Of the 32 compounds that did not yield fragment hits, 27 formed well diffracting crystals. The omit difference map is shown in the vicinity of the known benzamidine ligand (present in all crystals) for the best (A) and the worst (B) non fragment binding diffractors. Panel C shows the electron density from *in situ* co-crystallization of trypsin with imidazole (PDB 4NCY), an unreported hit for this screened condition (difference omit maps are contoured at 3σ).

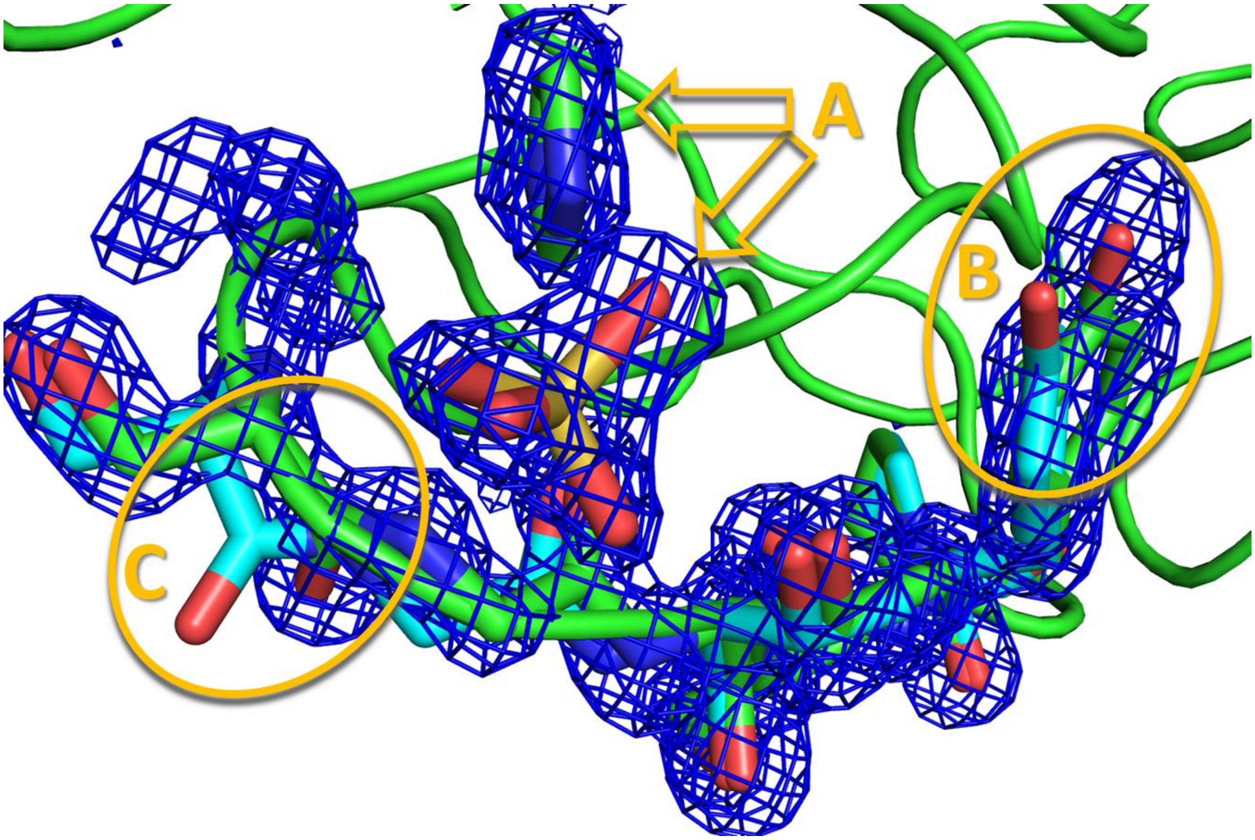

Supporting Figure 2: Imidazole binds to a location far from the trypsin active site after *in situ* co-crystallization. Electron density is contoured at 3σ for a difference omit map using data from the trypsin co-crystallized with imidazole (the ligands and all moving residues were excluded from the omit, shown in green). The native structure is shown for comparison (shown in blue). The imidazole prompts an SO_4^- anion to bind simultaneous (A). This in turn induces a significant conformational change in a five residue loop region from S146 to Y151. Tyrosine 151 moves away from the SO_4^- anion (via a water bridge)(B), while the serine 147 backbone rotates in towards the SO_4^- (C).

Supporting references

Cheng, Y., Judd, T. C., Bartberger, M. D., Brown, J., Chen, K., Fremeau, R. T. Jr & Wood, S. (2011). *J. Med. Chem.* **54**, 5836-5857.

Rees, D. C., Congreve, M., Murray, C. W. & Carr, R. (2004). *Nature Rev. Drug Discov.* **3**, 660-672.

Trott, O. & Olson, A. J. (2010). J. Comput. Chem. **31**, 455-461.
